# Inter-tumor genomic heterogeneity of breast cancers: comprehensive genomic profile of primary early breast cancers and relapses

**DOI:** 10.1186/s13058-020-01345-z

**Published:** 2020-10-15

**Authors:** Caterina Fumagalli, Alberto Ranghiero, Sara Gandini, Federica Corso, Sergio Taormina, Elisa De Camilli, Alessandra Rappa, Davide Vacirca, Giuseppe Viale, Elena Guerini-Rocco, Massimo Barberis

**Affiliations:** 1 Division of Pathology and Laboratory Medicine, IEO, European Institute of Oncology, IRCCS, Milan, Italy; 2 Division of Epidemiology and Biostatistics, IEO, European Institute of Oncology IRCCS, Milan, Italy; 3 Department of Oncology and Hemato-Oncology, University of Milan, Milan, Italy

**Keywords:** Breast cancer, Recurrence, Genomic heterogeneity, *TP53*, *MYC*, Next-generation sequencing, Comprehensive genomic profile

## Abstract

**Background:**

The breast cancer genome dynamically evolves during malignant progression and recurrence. We investigated the genomic profiles of primary early-stage breast cancers and matched relapses to elucidate the molecular underpinnings of the metastatic process, focusing on potentially actionable alterations in the recurrences.

**Methods:**

A mono-institutional cohort of 128 patients with breast cancers (*n* = 68 luminal B HER2, *n* = 6 luminal B HER2+, *n* = 1 HER2+ non-luminal, *n* = 56 triple negative) and at least one recurrence in a timeframe of 17 years was evaluated. Next-generation sequencing comprehensive genomic profiling was performed on 289 formalin-fixed paraffin-embedded (FFPE) samples, including primary tumors and matched relapses. Correlations of genomic aberrations with clinicopathologic factors and time to breast cancer relapse were analyzed.

**Results:**

Genomic data were available for 188 of 289 FFPE samples that achieved the sequencing quality parameters (failure rate 34.9%), including 106 primary tumors and 82 relapses. All primary and relapse samples harbored at least one genomic alteration, with a median number of six alterations per sample (range 1–16). The most frequent somatic genomic alterations were mutations of *TP53* (primary tumors = 49%, relapses = 49%) and *PIK3CA* (primary tumors = 33%, relapses = 30%). Distinctive genomic alterations of primary tumors were significantly associated with molecular subtypes. *TP53*, *PIK3R1*, and *NF1* somatic alterations were more frequently detected in triple negative tumors (*p* value < 0.05); *CCND1*, *FGF3*, and *FGFR1* copy number gains were recurrently identified in luminal cases (*p* value < 0.05). Moreover, *TP53* mutations and *MYC* amplification were significantly and independently associated with a shorter time to relapse (*p* value < 0.05). Molecular subtype changes between primary tumors and relapses were seen in 10 of 128 (7.8%) cases. Most driver genomic alterations (55.8%) were shared between primary tumors and matched recurrences. However, in 39 of 61 cases (63.9%), additional private alterations were detected in the relapse samples only, including 12 patients with potentially actionable aberrations.

**Conclusions:**

Specific genomic aberrations of primary breast cancers were associated with time to relapse. Primary tumors and matched recurrences showed a core of shared driver genomic aberrations but private actionable alterations have been identified in the relapses.

## Background

Breast cancer is the most commonly diagnosed cancer and the leading cause of tumor-related mortality in women worldwide [1]. Nearly 20–30% of patients with early-stage breast cancer experience local or distant recurrence even after standard loco-regional and adjuvant treatments [2]. Although great efforts have been made to identify prognostic biomarkers for risk stratification [3–6], the biological underpinnings of the recurrence are still poorly characterized, and predictive biomarkers leading to individualized treatments in the recurrence setting are still needed.

Large-scale next-generation sequencing technologies have provided valuable insights into the genomic landscape of breast cancers. The most recurrent alterations affect *PIK3CA* and *TP53* genes. *CCND1* copy number gain is more frequent in estrogen receptor (ER)-positive breast cancer. *MYC* amplification and homologous recombination deficiency, including *BRCA1* pathogenic variants, have been reported more frequently in triple negative (ER−/PR−/HER2−) tumors [7, 8]. *FGFR1* amplification has been associated with poor prognosis in hormone receptor-positive, lymph node-positive breast cancers [9]. *HER2* amplification [10] or *PIK3CA* and *ESR1* mutations [11–13] have been linked to response to therapy. However, a heterogeneous spectrum of driver alterations characterizes the molecular portrait of invasive breast tumors and their clinical implications remain to be fully elucidated.

The dynamic evolution of the breast cancer genome from pre-invasive stages to metastasis is ruled by phenomena of spatial and temporal heterogeneity [14]. Spatial heterogeneity can involve distinct areas within a tumor causing differences at morphologic, genomic, transcriptomic, and proteomic levels. Temporal heterogeneity indicates the variations between primary and metastasis caused by the metastatic process itself as well as the therapeutic interventions administered. Therefore, given the intra-tumor genetic heterogeneity, a clonal selection event, as well as the onset of additional alterations, may occur during tumor evolution over time or in response to therapy.

In this study, we analyzed a single-institution cohort of 128 patients with early-stage breast cancer and at least one (regional or distant) recurrence in 17 years. We performed comprehensive genomic profiling of primary and matched relapsed tumors aiming to (i) define the repertoire of genetic alterations of primary and metastatic/recurrent breast cancers and (ii) their association with specific clinico-pathological features and (iii) identify additional and potentially actionable alterations in the metastasis/recurrence site.

## Methods

### Study population

The study population included a mono-institutional cohort of 128 patients with early-stage breast cancer that underwent surgery at the European Institute of Oncology of Milan between 1999 and 2019. All the patients had at least one loco-regional or distant relapse in a timeframe of 17 years. The median age at diagnosis was 50 years (range 24–79 years). The median time to relapse was 42.5 months, range 1–200 months. Primary and relapsed tumors were classified according to immunohistochemical surrogates, following the St. Gallen criteria [15]. Estrogen receptor and progesterone receptor (PR), HER2, and proliferation index (Ki-67) were evaluated by immunohistochemistry, and for HER2 equivocal cases (immunohistochemical score 2+), reflex fluorescence in situ hybridization (FISH) analysis was performed. The tumors were classified as luminal B HER2− (hormone receptors+/HER2−), luminal B HER2+ (hormone receptors+/HER2+), HER2+ non-luminal (hormone receptors−/HER2+), and triple negative (hormone receptors−/HER2−). The recurrence sites were divided into loco-regional (axillary lymph-node, skin or soft tissue of chest wall) or distant (liver, lung, distant lymph node, pleura, distant soft tissue, ovary, bone) relapses. The clinico-pathological characteristics of the study population are reported in Table 1. All patients gave written informed consent regarding the storage of any biological specimens collected in the course of diagnosis and the use of these samples for research purposes. The study was conducted in accordance with the 1964 Helsinki Declaration and later amendments.

**Table 1:** Clinico-pathological characteristics of the study population

Clinico-pathological features		
**Primary tumors (*n*** = 133*)		***N*** **(%)**
**Subtype**	IBC, NOS	114 (85.7%)
	Lobular	7 (5.3%)
	Other special types	7 (5.3%)
	Mixed	5 (3.8%)
**Molecular subtype (IHC surrogates)**	Luminal B HER2−	68 (51.1%)
	Luminal B HER2+	6 (4.5%)
	HER2+ non-luminal	1 (0.8%)
	Triple negative	58 (43.6%)
**pT**	1b	9 (6.7%)
	1c	43 (32.3%)
	2	55 (41.4%)
	3	17 (12.8%)
	4	4 (3%)
	NA	5 (3.8%)
**pN**	0	34 (25.6%)
	1–3	80 (60.2%)
	NA	19 (14.3%)
**Recurrences/metastasis (*n*** = 135**)		***N*** **(%)**
**Site**		
Local (*n* = 47)	Axillary lymph node	24 (51.1%)
	Skin	14 (29.8%)
	Soft tissue	9 (19.1%)
Distant (*n* = 88)	Bone	2 (2.3%)
	Liver	22 (25%)
	Lung	14 (15.9%)
	Lymph node	4 (4.5%)
	Ovary	2 (2.3%)
	Pleura	37 (42%)
	Skin	3 (3.4%)
	Soft tissue	4 (4.5%)

The study populations included 128 women affected by breast cancer and relapsed in a timeframe of 17 years

*IHC* immunohistochemistry, *pT* pathologic stage classification of primary tumor, *pN* pathologic stage classification of regional lymph nodes, *IBC* invasive breast carcinoma, *NA* not available

*N = 133, 5 patients had multiple primary tumors

**N = 135, 6 patients had multiple recurrences

### Comprehensive genomic profiling of primary tumors and relapses

Primary tumors and recurrences were evaluated using large multi-genes NGS panels detecting different types of genetic alterations such as single nucleotide variants (SNVs), insertion/deletions (InDels), copy number variants (CNVs), and fusion genes. Representative formalin-fixed paraffin-embedded (FFPE) tumor tissue blocks of primary tumors and recurrences were retrieved from the archives of the Division of Pathology of the European Institute of Oncology. In the first phase of the study, 202 FFPE blocks were analyzed with the Foundation One test, including 315 genes (Roche Pharma AG, Grenzach-Wyhlen, Germany). We then performed a NGS panel in-house (Oncomine Comprehensive Assay v.3, ThermoFisher Scientific, Waltham, MA, USA), for 87 additional samples, following the manufacturer’s instructions. Briefly, the nucleic acids (DNA and RNA) were extracted automatically using Promega Maxwell RSC DNA or RNA FFPE kit (Promega, Madison, WI, USA) and then quantified, as previously reported [16]. Ten nanograms of genomic DNA and 10 ng of RNA were used for the library preparation and the subsequent chip loading, both performed automatically on the Ion Chef System (ThermoFisher Scientific, Waltham, MA, USA). The sequencing run was done on Ion S5 System (ThermoFisher Scientific, Waltham, MA, USA) and data were analyzed using the Ion Reporter Analysis Software v5.10. Only mutations with a variant allele frequency (VAF) equal/superior to 5% and with adequate quality metrics (read depth > 100; VAF × read depth > 25; *p* value = 0.00001) were reported. Copy number variants were evaluated for samples with a Median of the Absolute values of all Pairwise Difference (MAPD) < 0.5 [17].

The mutations were classified as *driver alterations*, including all the alterations belonging to level I, II, and III class, as previously described [18] or *variants of uncertain significance (VUS)* if they were annotated as *unknown* in cancer gene mutation databases, including Catalogue of Somatic Mutations in Cancer (COSMIC) [19], cBioPortal for Cancer Genomics [20], and ClinVar–NCBI–NIH [21], or considered damaging by “in silico” predictors only, available at VarSome website [22]. Furthermore, we evaluated the clinical actionability of driver alterations using OncoKB levels of evidence V2 ranking [23]. Variants classified as polymorphism, benign, likely benign, or neutral were not reported. The co-occurrence of selected gene alterations was evaluated using the mutual exclusivity analysis of cBioPortalbioinformatics tool [20].

For this analysis, only alterations occurring in genes targeted by both the FoundationOne and Oncomine Comprehensive Assays (Additional file 1 for the complete gene list) were evaluated. Overall, 188 of 289 (65.1%) samples analyzed with large NGS panels achieved the quality parameters required by the specific assay, including 106 primary and 82 recurrence tumors. Moreover, for 70 of 128 (54.7%) patients, both primary and matched relapse sample were successfully profiled with the same NGS panel.

### Statistical analysis

Patient clinico-pathological characteristics were reported with median and interquartile range (IQR) for continuous variables and absolute and relative frequencies for categorical variables. Cohen’s Kappa test was used to assess the driver gene alteration agreement between primary and relapse samples. Genes with a Kappa coefficient over 60% and a mutation frequency over 10% were considered in this analysis. Hamming distance for binary variables was applied to implement a heatmap of the selected driver genes. Univariate logistic models were used to assess the associations of gene aberrations with molecular subtype (triple negative vs luminal B) and recurrence site (distant vs loco-regional). The results were shown using the R package “EnhancedVolcano” for Volcano plot implementation. The association of driver gene alterations with time to recurrence was analyzed with a Log-rank test. We also evaluated the association of the total number of mutations in primary breast cancer samples as categorical variables, with the median value as cutoff. Multivariate Cox proportional hazard models were chosen considering backward and forward selection of variables and adjusting for known prognostic factors (molecular subtype and pT). Hazard ratios (HR) with 95% confidence intervals (95% CI), from multivariate models, were reported. Since the survival analyses were carried out only for patients with a relapse, hazard ratios represented a measure of the association with time to relapse and they should not be interpreted as probability of relapse. Molecular subtype changes between primary breast cancer and relapse samples were displayed in a Chord Diagram from the R package “circlize.” Finally, Fisher exact tests were employed to estimate whether gene aberrations in primary and recurrence samples were significantly different by molecular subtype (triple negative vs luminal B). Only gene alterations with a frequency over 5% in both primary breast and relapse groups were used in this analysis. Due to the explorative nature of this study, a multiplicity correction was omitted.

## Results

### Molecular portraits of primary early breast cancers and correlations with clinico-pathological characteristics

Overall, 106 primary tumors were analyzed, including 56 (52.8%) luminal B HER2−, 45 (42.5%) triple negative, 4 (3.8%) luminal B HER2+, and 1 (0.9%) HER2+ non-luminal. All primary samples harbored at least one gene alteration (driver or VUS), with a median number of six alterations per sample (range 1–16). Driver alterations were detected in 102 of 106 (96%) samples, with a median number of three driver alterations per sample (range 0–10). In detail, we found 721 gene aberrations (driver or VUS), including 472 (65.5%) SNV, 75 (10.4%) InDels, 162 (22.5%) CNV, and 12 (1.7%) fusion genes (Additional file 2). 352/721 (48.8%) were driver alterations, including 135 (38.4%) SNV, 51 (14.5%) InDels, 160 (45.5%) CNV, and 6 (1.7%) fusion genes (Additional file 2).

**Fig. 1:**
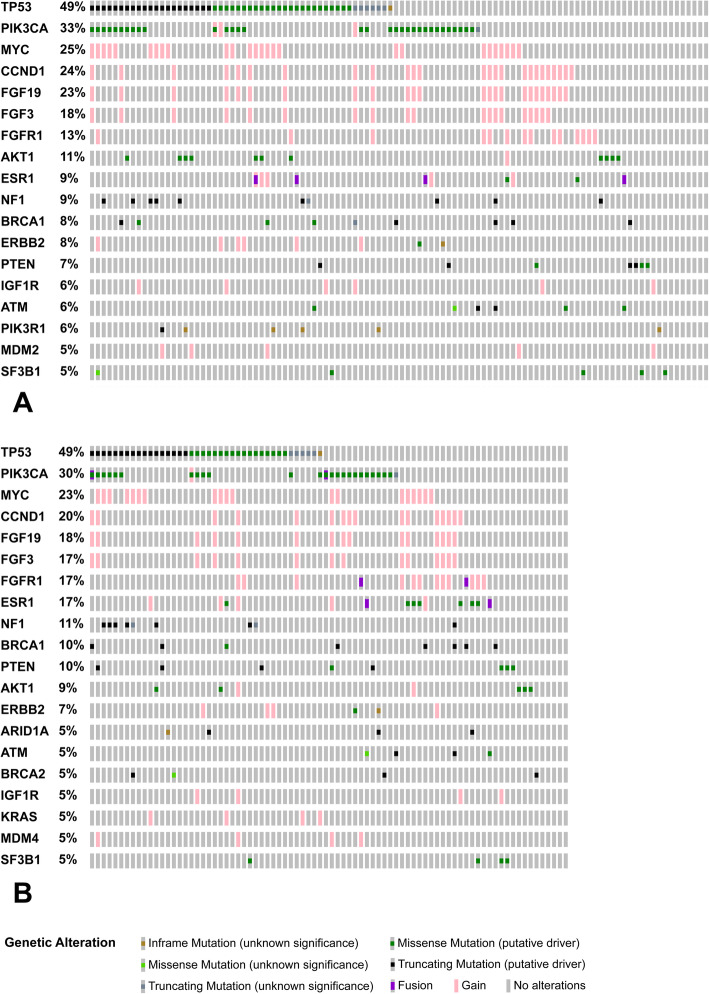
Distribution and co-occurrence of recurrent driver genomic alterations. Oncoprint plots showed genes altered in more than 5% of breast cancers samples. **a** Primary tumors (*n* = 106). **b** Recurrences (*n* = 82). Each gene was reported in rows; each case was reported in columns. Significant co-occurrent and recurrent copy number gains involved *CCND1*, *FGF19*, and *FGF3* genes (*p* value < 0.001 and *q* < 0.01, according to mutual exclusivity analysis). Oncoprinter tool - cBioportal (https://www.cbioportal.org/oncoprinter) was used to create graphs and perform mutual exclusivity analysis

The most frequently mutated genes were *TP53* and *PIK3CA* (Fig. 1a), with alterations spanning the whole coding sequence of *TP53* and involving hotspot regions in *PIK3CA* (Additional file 3). Moreover, recurrent copy number gains were identified in *MYC*, *CCND1*, *FGF19*, *FGF3*, and *FGFR1* (Fig. 1a). Among them, *CCND1*, *FGF19*, and *FGF3* genes mapped on the same cytogenetic band, 11q13.3, and showed a statistically significant co-occurrence (*p* value < 0.001 and *q* < 0.01). No other significant driver alterations co-occurrence was found. Distinctive genomic alterations were significantly associated with molecular subtypes (Additional files 4 and 5). In particular, *TP53*, *PIK3R1*, and *NF1* mutations were detected more frequently in triple negative tumors (odds ratio > 2.71, *p* value < 0.05). *CCND1*, *FGF3*, and *FGFR1* copy number gains were recurrently identified in luminal cases (odds ratio < 0.36, *p* value < 0.05) (Additional file 4).

**Fig. 2:**
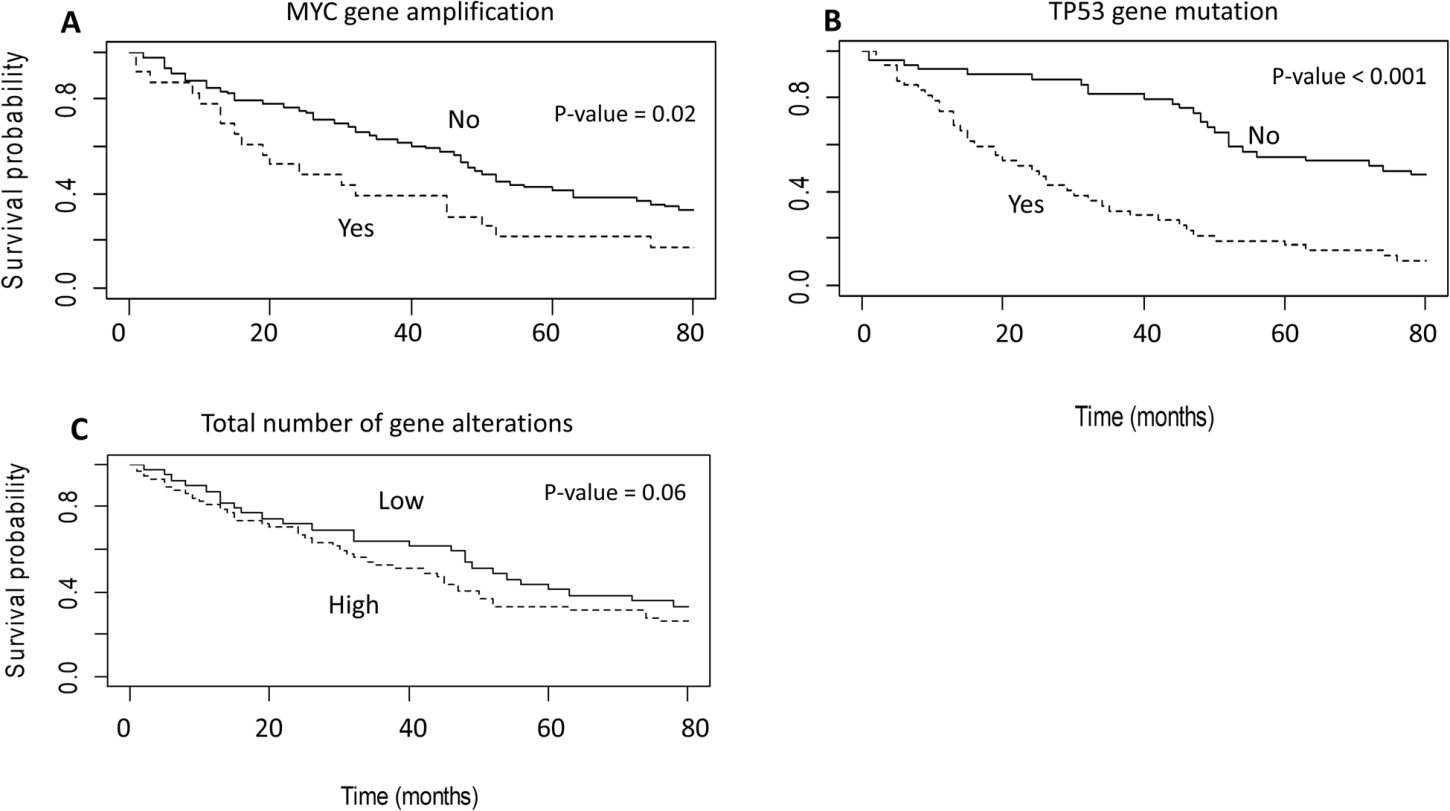
Kaplan-Meier (Log-rank test) curves of disease-free survival according to molecular alterations in primary tumors. The presence of *MYC* copy number gain (**a**) and *TP53* mutations (**b**) in primary tumors were significantly associated with a shorter time to relapse (*p* value < 0.05). A trend of association was observed between a higher number of genomic alterations (**c**) and a shorter time to relapse (*p* value 0.06). The median value (*n* = 6) of alterations per primary tumor sample was used as a cut-off to define low and high number of alterations

Moreover, *TP53* mutations and *MYC* copy number gain were significantly associated with shorter time to relapse, both in univariate and multivariate analyses, adjusted for known prognostic factors (*p* value < 0.05) (Fig. 2, Table 2). Also, an increased number of alterations, including both driver and VUS variants, was associated with a shorter time to relapse in univariate analysis, even if not statistically significant (*p* value 0.06, Fig. 2).

**Table 2:** Multivariable Cox proportional hazard model showing gene alterations associated with time to relapses

Variables	Contrast	HR	Low 95	Up 95	***p*** value
*TP53 mutations*	Yes vs no	1.85	1.07	3.21	0.02
*MYC copy number gain*	Yes vs no	1.71	1.01	2.92	0.04
pT	2–4 vs 1	2.41	1.49	3.89	< 0.001
Molecular subtypes	TN vs others	2.34	1.36	4.04	0.002

*HR* hazard ratio, *TN* triple negative

*TP53* mutations and *MYC* copy number gain findings in primary breast cancers were prognostic factors independently associated with shorter time to relapse, along with a high primary tumor pathologic stage (pT = 2–4) and triple negative molecular subtype.

### Molecular portraits of recurrences and correlations with clinico-pathological characteristics

Molecular subtype changes between primary tumors and relapses were seen in 10 of 128 (7.8%) cases (Additional file 6). In detail, 5 patients with luminal B HER2− primary tumors had triple negative recurrences, 3 patients with triple negative primary tumor relapsed with luminal HER2− tumor, and 2 patients with luminal HER2+ primary disease had a luminal HER2− recurrence. All the 82 recurrence samples that underwent a comprehensive genomic profile harbored at least one alteration, with a median number of six alterations per sample (range 1–16). Driver alterations were identified in 78 of 82 (95.1%) samples, with a median number of three driver alterations per sample (range 0–11). Overall, in the recurrence samples, we found 549 aberrations (driver and VUS), including 352 SNV (64.1%), 59 InDels (10.7%), 126 CNV (23%), and 12 fusion genes (2.2%). Considering the driver alterations only (*n* = 291; 53%), we detected 112 (38.5%) SNV, 46 (15.8%) InDels, 125 (43%) CNV, and 8 (2.7%) fusion genes. A higher number of driver alterations, including SNVs, CNVs, InDels, and fusion genes, were seen in the relapses as compared to the primary tumors, although not statistically significant (Additional file 2).

As in the primary tumors group, the most frequently mutated genes were *TP53* and *PIK3CA* (Fig. 1b), with alterations spanning the whole coding sequence of *TP53* and involving hotspot regions in *PIK3CA* (Additional file 3). An increased frequency of alterations in a subset of genes was seen in the recurrence (R) as compared to primary (P) samples, including *FGFR1* (13% P–17% R), *ESR1* (9% P–17% R), *NF1* (9% P–11% R), *BRCA1* (8% P–10% R), and *PTEN* (7% P–10% R), even if not statistically significant (Fig. 1).

Similarly to the primary tumors, specific alterations identified in the recurrence samples were associated with molecular subtypes (Additional files 4 and 5), including *TP53* and *NF1* alterations in triple negative tumors (odds ratio > 2.71 *p* value < 0.05) and *CCND1*, *FGFR1*, and *ESR1* aberrations in luminal cases (odds ratio < 0.36 *p* value < 0.05).

### The evolution of genomic landscape between primary and matched breast cancer relapses

**Fig. 3:**
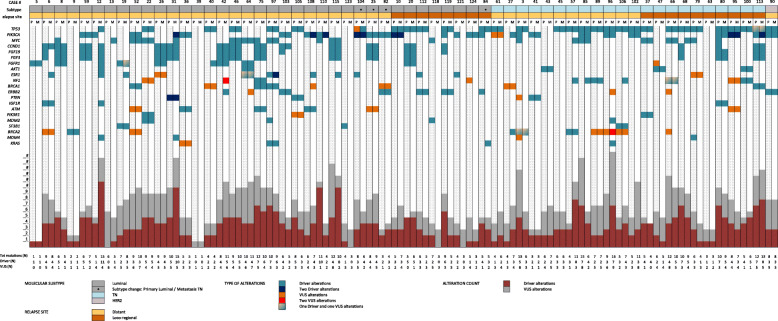
Distribution of driver and VUS alterations in 61 matched primary tumors and relapses. Each column represents one case, with primary tumors in blank columns and matched relapses in dashed columns. The cases were grouped according to the molecular subtype (luminal B, triple negative or HER2-non luminal) and the recurrence site (distant vs loco-regional) color-coded as in the legend. The most frequently altered genes (occurring in more than 5% of samples) and the type of alterations (driver or VUS) were reported in rows and color-coded according to the legend. The total number of alterations affecting each sample was shown in the lower part of the figure

**Fig. 4:**
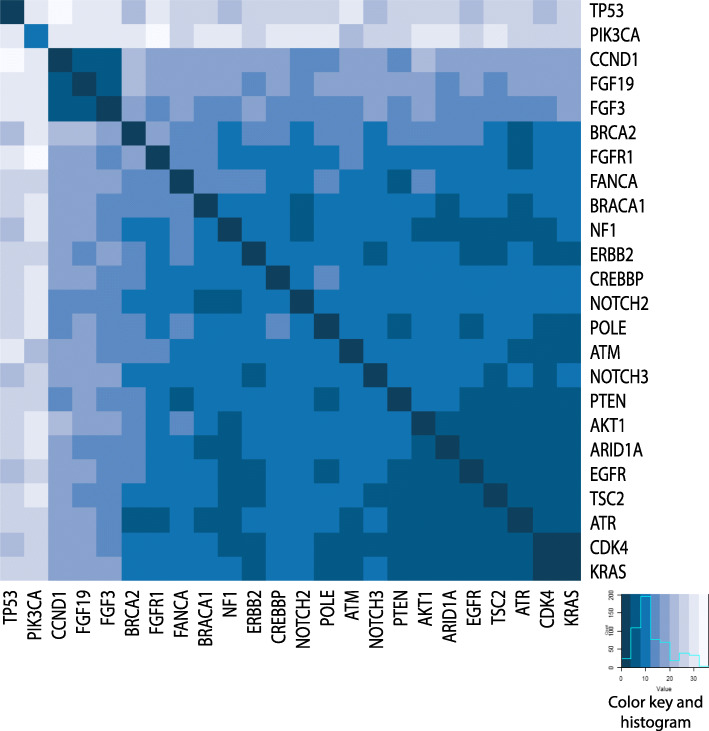
Concordance of the driver genetic alterations identified in primary tumors and matched relapses. Only genes altered in more than 10% of the study population and with a concordance of at least 60% between matched primary tumor and relapse were reported in the heatmap, with primary tumors on *X*-axis and matched recurrence samples on *Y*-axis. Darker blue color indicated a higher level of gene driver alteration concordance. The recurrent co-occurrence of copy number gains of *CCND1*, *FGF19*, and *FGF3* genes was pin-pointed

In 70 cases, we successfully analyzed primary tumors and matched recurrences with the same NGS panel. Among these, 61 patients had a single primary and relapse specimen (Fig. 3). We found that 55.8% of driver alterations were shared between primary tumors and recurrences, with a median number of two aberrations in common per sample (range 0–7). Including the variants of unknown significance, the prevalence of shared alterations was 61.2% (median = 5; range 0–11) (Additional file 7). Moreover, the most recurrent driver alterations identified in the primary samples were maintained in the recurrence, including mutations of *TP53* and *PIK3CA* and copy number gain of *CCND1*, *FGF19*, *FGF3*, and *FGFR1* (Fig. 4). The number of shared aberrations was significantly associated with the time to relapse. Considering the median relapse time (50 months) of the cases successfully profiled, those with early and late recurrences showed a different proportion of shared aberrations (66.7% versus 56%, respectively) and driver (62.9% versus 49.3% respectively) alterations (Additional file 8). Indeed, in 39 of 61 patients (63.9%), additional private alterations were detected in the recurrence samples only, affecting breast cancer-related genes as *ERBB2*, *ESR1*, *FGFR1*, or *NF1*, and including 12 patients (19.7%) with clinically relevant alterations according to the OncoKB levels of evidence V2 ranking (levels 1–3) (Additional files 9 and 10). Nine cases had multiple primary and/or recurrence samples available for the analysis (Additional file 11). Although spatial and temporal heterogeneity was seen, most driver alterations were retained in the different recurrence samples.

## Discussion

In the present study, we performed a comprehensive genomic profile of 106 primary breast cancers and 82 recurrences, including 70 cases with matched primary and relapse samples. We identified specific molecular characteristics of primary tumors associated with time to relapse. Moreover, we showed that a backbone of recurrent driver molecular alterations of primary tumors was retained in the recurrences. However, additional private genomic aberrations were detected in relapse samples, including clinically relevant genes and potentially actionable targets.

Overall, more than 95% of the specimens under investigations, including both primary and recurrences, harbored at least one driver mutation, with a median number of three driver alterations per sample. As previously reported in larger series [24, 25], we found heterogeneous genomic profiles, with few recurrent molecular aberrations, including mutations of *TP53* and *PIK3CA* and copy number gains *MYC*, *CCND1*, *FGF19*, *FGF3*, and *FGFR1*. Specific alterations were significantly associated with breast cancer subtypes in both primary tumors and recurrences. *TP53* and *NF1* mutations were more frequently identified in triple negative breast cancers whereas *CCND1*, *FGF3*, *FGF19*, *ESR1*, and *FGFR1* copy number gains were recurrent in tumors of luminal subtype. In this latter group, a significant association between *ESR1* alterations and recurrence was seen, probably reflecting the mechanism of acquired resistance to endocrine therapies [26].

*TP53* mutations and *MYC* copy number gains in primary tumors were significantly associated with the time to relapse. This association was retained in the multivariable analysis adjusted for known prognostic factors. The clinical value of somatic *TP53* mutations has been largely evaluated according to a specific mutation type, protein domain involved, gene expression data (i.e., PAM 50), and hormone receptor status [27–29]. Moreover, MYC deregulation plays a critical role in cell proliferation and tumor progression, and it has been associated with an aggressive clinical behavior and poor prognosis in breast cancer [30–35]. As previously reported [36], our data suggest that *MYC* and *TP53* alterations may represent independent poor prognostic factors in early-stage breast cancer. Moreover, an increased number of total mutations in primary tumors could be associated with a shorter time to relapse. Even if different NGS panels have been used in this study and the data should be further validated, these findings are consistent with the observation that a high tumor mutation burden may correlate with a poor prognosis in various cancer types [37–39].

To focus on the dynamic evolution of breast cancer genome, we firstly compared the overall data between primary and recurrence tumors. A trend of an increasing number of driver alterations of breast cancer-related genes was observed in recurrence samples as compared to primary tumors, including alterations in *FGFR1*, *ESR1*, *NF1*, *BRCA1*, and *PTEN* genes. These data may have a clinical impact since the additional burden of alterations included actionable or druggable genes [13, 40, 41] or genes related to therapy resistance. In particular, *NF1* alterations have been reported in association with endocrine therapy resistance in lobular breast cancer [42]. Recently, Pearson et al. showed that *NF1* mutations were frequently acquired in breast cancer at progression and were associated with shorter survival in hormone receptor-positive breast cancers relapsing during adjuvant endocrine therapy [43]. However, in our series, mutations of *NF1* were more frequently detected in tumors of triple negative subtype. Further studies are needed to confirm these findings and to unveil the biological and clinical significance of this alteration in triple negative breast cancers.

We evaluated in detail the evolution of breast cancer biology at the single patient level with the analysis of matched primary and relapse samples. Molecular subtype changes from primary tumors to recurrences were seen in 7.8% of cases. These proportions were lower but consistent with previously reported meta-analysis data of larger cohorts [44–51]. We observed a high level of concordance (55.8%) of genomic aberrations between primary and matched relapse specimens. However, the proportion of shared aberrations was lower in cases with later recurrence (49.3%). Indeed, Yates and colleagues showed that the number of mutations was similar in primary and synchronous metastasis but a high number of mutations accumulated during breast cancer evolution and can be detected in samples from late relapses [52]. Moreover, 63.9% of cases had private alterations in their recurrence, including 19.7% of patients with clinically actionable aberrations (e.g., affecting *ERBB2*, *BRCA2*, *PIK3CA*) that may be targeted by available biological drugs [23].

This study has several limitations. First, given the failure rate of NGS performed on nucleic acids extracted from old FFPE samples, we were able to evaluate only 65.1% of the cases. As known, stringent NGS quality metrics are needed to obtain robust results when long-term stored FFPE specimens are investigated. In our cohort, 73 (25.3%) specimens had an archival time longer than 10 years, and 79 (27.3%) cases longer than 5 years. Second, using two different NGS panels (FoundationOne and Oncomine Comprehensive Assay), only data about genes included in both panels were considered and only matched primary tumors and recurrences investigated with the same panel could be analyzed. Third, given the retrospective nature of this study including a heterogeneous cohort of patients with breast cancer, we were unable to perform detailed survival analysis or to test the effect of therapy on molecular alterations acquired at progression. Finally, this is an exploratory hypothesis-generating study evaluating the genomic profile of primary breast cancers and breast cancer relapses to investigate inter-tumor genomic heterogeneity. Although the most recurrent driver alterations of primary tumors were detected in matched relapses, we showed that additional and potentially actionable alterations may be detected in the recurrence sample only, as showed by OncoKB levels assessment. Given that we analyzed samples from a retrospective cohort of patients, we could not assess the clinical impact of these findings. However, our data may suggest that inter-tumor genomic heterogeneity of breast cancers might be of clinical relevance and the genomic profile of breast cancer relapses might guide patients tailored treatments. Further ad hoc investigation is needed to confirm our findings and evaluate their clinical impact.

## Conclusions

In conclusion, this study showed that the presence of *TP53* mutations and *MYC* copy number gain in primary early-stage breast cancers were independently associated with time to relapse. A trend of association between the number of genomic alterations and time to relapse was seen and required further investigation. Although shared driver aberrations were identified in primary tumors and matched recurrences, comprehensive genomic profiling of relapse samples may reveal additional private and actionable alterations.

## Supplementary information

**Additional file 1: Supplementary Table 1**.docx: Genes and type of alterations investigated in both FoundationOne and Oncomine Comprehensive Assay panels.

**Additional file 2: Supplementary Figure** 1.jpg: Gene alterations distribution according to type and pathogenic significance (driver or VUS). A) Primary tumors (*n* = 106); B) Recurrences (*n* = 82). SNV: Single Nucleotide Variants; INDEL: Insertions/Deletions; CNV: Copy Number Gain; VUS: Variants of Uncertain Significant; LUM: non-TN tumor; TN: triple negative tumor.

**Additional file 3: Supplementary Figure 2**.jpg: Lollipop plots of mutations distribution on *TP53* and *PIK3CA* proteins. A) Primary tumors (*n* = 106), B) Recurrences (*n* = 82). Each mutation is represented by single lollipop; the stick lengths indicate mutation frequency (y-axis), and dots are color-coded according to alteration type: green, missense mutations; black, truncating mutations (frameshift or nonsense mutations); brown dots, in-frame mutations. Graphs created using Mutation Mapper tool, cBioportal (http://www.cbioportal.org/mutation_mapper) and manually curated. TP53: RefSeq: NM_000546; Ensembl: ENST00000269305; CCDS:CCDS11118; UniProt: P53_HUMAN; PIK3CA: RefSeq:NM_006218, Ensembl: ENST00000263967; CCDS:CCDS43171; UniProt: PK3CA_HUMAN.

**Additional file 4: Supplementary Figure 3**.jpg: Volcano plots of the distribution of gene driver alterations according to molecular subtype. A) Primary tumors (*n* = 106), B) Relapses (*n* = 82). LUM: non-TN tumor; TN: triple negative tumor. Green dot: genes differentially affected by alteration according to OR (OR < 0.36 or > 2.71;); Red dot: genes differentially affected by alteration according to OR and *p* value (OR < 0.36 or > 2.71; p value < 0.05). OR: Odds Ratio.

**Additional file 5: Supplementary Figure 4**.jpg: Molecular alterations found in primary tumor and recurrent samples, according to molecular subtype. A) Primary tumors (*n* = 106), B) Relapses (*n* = 82).

**Additional file 6: Supplementary Figure 5**.jpg: Chord diagram representing molecular subtype change (IHC surrogates) between primary tumors and matched relapses.

**Additional file 7: Supplementary Figure 6**.jpg: Venn diagrams of shared and private genomic alterations between paired primary tumor and relapse samples. For each case (*n* = 61) primary tumor and matched recurrence were compared, considering unique and shared alterations. A) Driver alterations only, B) Driver and VUS alterations.

**Additional file 8: Supplementary Figure 7**.jpg: Venn diagrams of shared and private genomic alterations between paired primary tumor and relapse samples according to time to relapse. For each case (*n* = 61) primary tumor and matched recurrence were compared, considering unique and shared alterations. The median time to relapse in the series (50 months) was considered as cut-off. A) Driver alterations only, B) Driver and VUS alterations.

**Additional file 9: Supplementary Figure 8**.jpg: Distribution of gene with additional driver alterations identified in recurrence samples only. Genes with additional alterations in the relapse samples and the number of additional private alteration per gene were reported on X-axis and Y-axis, respectively. Each private alteration was color-coded according to OncoKB levels as reported in the legend. Blue box: no OncoKB level associated with the specific driver alteration.

**Additional file 10: Supplementary Table 2**.docx: Additional alterations found in the recurrence samples only. Bold: driver alterations.

**Additional file 11: Supplementary Figure 9**.jpg:: Graphical representation of 9 cases with multiple primary tumors and/or matched relapses. Each tree represented an individual patient. The hormone receptors status was reported for each primary tumor and relapse and color-coded according to the legend. Moreover, TNM staging and site were also indicated for each primary tumor and relapse, respectively. The time to relapse was depicted as a bar. Molecular findings were reported for each sample and color-coded as represented in the legend. Gene alterations shared between all the samples of the same patients were labeled with * mark.

## Data Availability

The datasets used and/or analyzed during the current study are available from the corresponding author on reasonable request.

## Abbreviations

NGS: Next-generation sequencing
P: Primary tumors
M: Metastases/relapses
ER: Estrogen receptor
PR: Progesterone receptor
IHC: Immunohistochemistry
SNV: Single nucleotide variants
InDels: Insertion/deletions
CNV: Copy number variants
FFPE: Formalin-fixed paraffin-embedded
VAF: Variant allele frequency
MAPD: Median of the Absolute values of all Pairwise Difference
VUS: Variants of uncertain significance
IQR: Interquartile range
HR: Hazard ratios
CI: Confidence intervals
