# Supplementary material for: Inter-tumor genomic heterogeneity of breast cancers: comprehensive genomic profile of primary early breast cancers and relapses
Source: Breast Cancer Res. 2020 Oct 15;22:107. doi: 10.1186/s13058-020-01345-z (PMC7566144; doi:10.1186/s13058-020-01345-z)
Supplement: Supplementary file 1 — Additional file 1: Supplementary Table 1.docx: Genes and type of alterations investigated in both FoundationOne and Oncomine Comprehensive Assay panels. [file 13058_2020_1345_MOESM1_ESM.docx]

**Additional file 1 - Supplementary Table 1:** Complete list of genes and type of alterations, investigated in both the FoundationOne and Oncomine Comprehensive Assay panels.

| **SNV, INDEL** | **SNV, INDEL** | **SNV, INDEL** | **CNV** | **FUSION** |
| --- | --- | --- | --- | --- |
| AKT1 | GNAS | PPP2R1A | AKT1 | ALK |
| AKT2 | H3F3A | PTCH1 | AKT2 | BRAF |
| AKT3 | HNF1A | PTEN | AKT3 | BRCA1 |
| ALK | HRAS | PTPN11 | ALK | BRCA2 |
| AR | IDH1 | RAC1 | AR | EGFR |
| ARAF | IDH2 | RAD51 | AXL | ETV4 |
| ARID1A | JAK1 | RAD51B | BRAF | ETV5 |
| ATM | JAK2 | RAD51C | CCND1 | FGFR1 |
| ATR | JAK3 | RAD51D | CCND2 | FGFR2 |
| ATRX | KDR | RAF1 | CCND3 | FGFR3 |
| AXL | KIT | RB1 | CCNE1 | MYB |
| BAP1 | KRAS | RET | CDK4 | NTRK1 |
| BRAF | MAP2K1 | RNF43 | CDK6 | NTRK2 |
| BRCA1 | MAP2K2 | ROS1 | CDKN2A | NUTM1 |
| BRCA2 | MAP2K4 | SETD2 | CDKN2B | PDGFRA |
| BTK | MAPK1 | SF3B1 | EGFR | RAF1 |
| CBL | MDM4 | SMAD4 | ERBB2 | RET |
| CCND1 | MED12 | SMARCA4 | ESR1 | ROS1 |
| CDK12 | MET | SMARCB1 | FGF19 | RSPO2 |
| CDK4 | MLH1 | SMO | FGF3 | TERT |
| CDK6 | MRE11A | SPOP | FGFR1 |  |
| CDKN1B | MSH2 | SRC | FGFR2 |  |
| CDKN2A | MSH6 | STAT3 | FGFR3 |  |
| CDKN2B | MTOR | STK11 | FGFR4 |  |
| CHEK1 | MYC | TP53 | FLT3 |  |
| CHEK2 | MYCN | TSC1 | IGF1R |  |
| CREBBP | MYD88 | TSC2 | KIT |  |
| CSF1R | NBN | U2AF1 | KRAS |  |
| CTNNB1 | NF1 | XPO1 | MDM2 |  |
| DDR2 | NF2 |  | MDM4 |  |
| EGFR | NFE2L2 |  | MET |  |
| ERBB2 | NOTCH1 |  | MYC |  |
| ERBB4 | NOTCH2 |  | MYCL |  |
| ESR1 | NOTCH3 |  | MYCN |  |
| EZH2 | NRAS |  | NTRK1 |  |
| FANCA | NTRK1 |  | NTRK2 |  |
| FBXW7 | NTRK2 |  | NTRK3 |  |
| FGFR1 | PALB2 |  | PDGFRA |  |
| FGFR2 | PDGFRA |  | PDGFRB |  |
| FGFR3 | PDGFRB |  | PIK3CA |  |
| FGFR4 | PIK3CA |  | PIK3CB |  |
| FLT3 | PIK3CB |  | PPARG |  |
| FOXL2 | PIK3R1 |  | RICTOR |  |
| GNA11 | PMS2 |  | TSC1 |  |
| GNAQ | POLE |  | TSC2 |  |
