# Supplementary figures and images for: Inter-tumor genomic heterogeneity of breast cancers: comprehensive genomic profile of primary early breast cancers and relapses
Source: Breast Cancer Res. 2020 Oct 15;22:107. doi: 10.1186/s13058-020-01345-z (PMC7566144; doi:10.1186/s13058-020-01345-z)

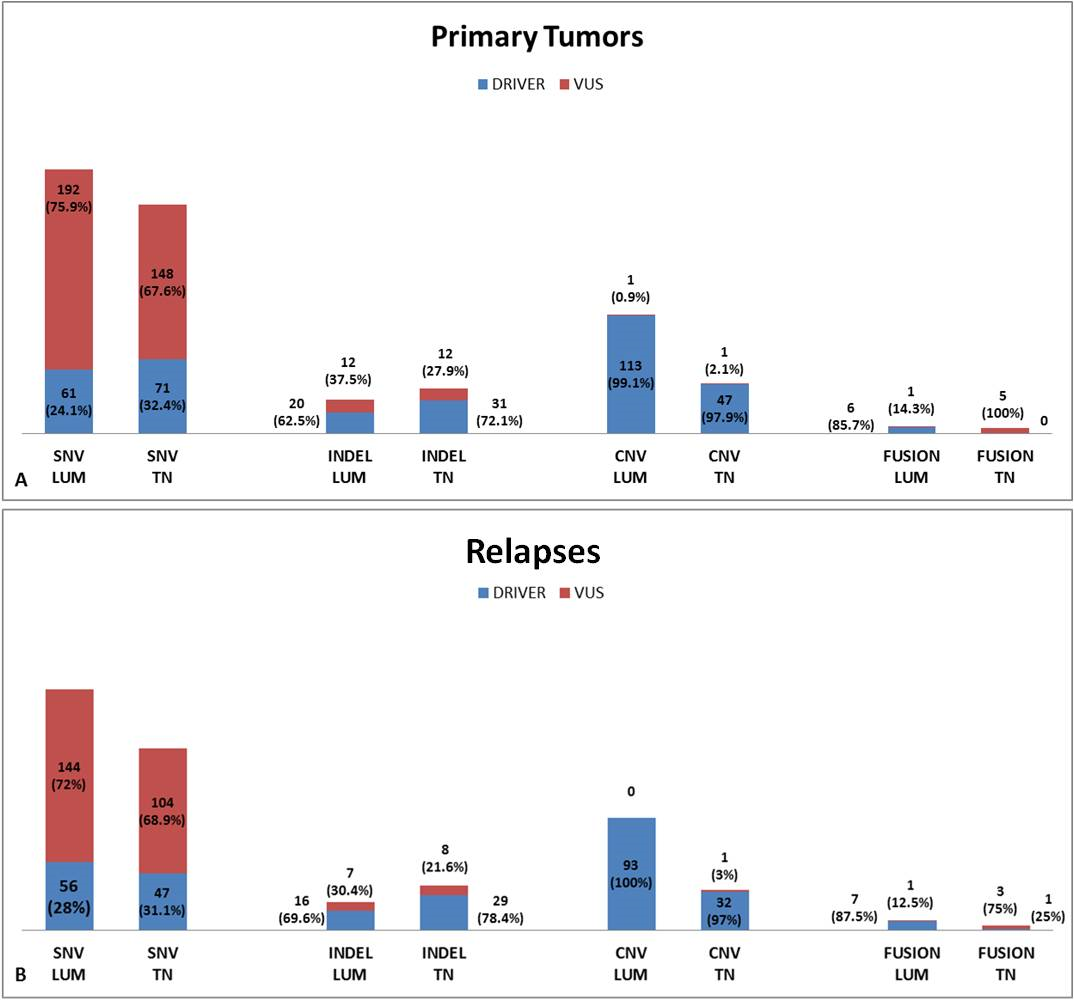

Supplement: Supplementary file 2 — Additional file 2: Supplementary Figure 1.jpg: Gene alterations distribution according to type and pathogenic significance (driver or VUS). A) Primary tumors (n = 106); B) Recurrences (n = 82). SNV: Single Nucleotide Variants; INDEL: Insertions/Deletions; CNV: Copy Number Gain; VUS: Variants of Uncertain Significant; LUM: non-TN tumor; TN: triple negative tumor. [file 13058_2020_1345_MOESM2_ESM.tif]

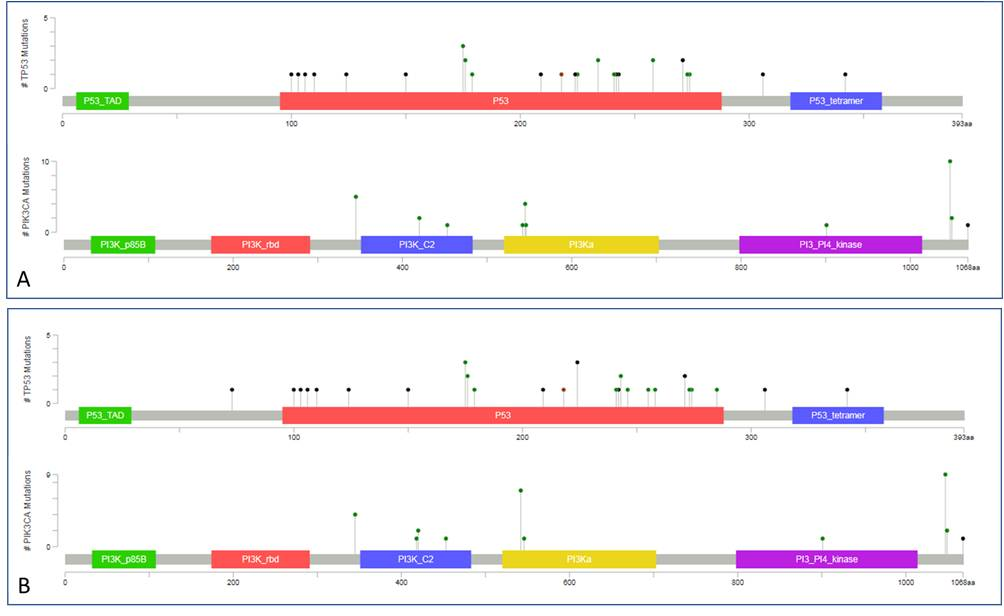

Supplement: Supplementary file 3 — Additional file 3: Supplementary Figure 2.jpg: Lollipop plots of mutations distribution on TP53 and PIK3CA proteins. A) Primary tumors (n = 106), B) Recurrences (n = 82). Each mutation is represented by single lollipop; the stick lengths indicate mutation frequency (y-axis), and dots are color-coded according to alteration type: green, missense mutations; black, truncating mutations (frameshift or nonsense mutations); brown dots, in-frame mutations. Graphs created using Mutation Mapper tool, cBioportal (http://www.cbioportal.org/mutation_mapper) and manually curated. TP53: RefSeq: NM_000546; Ensembl: ENST00000269305; CCDS:CCDS11118; UniProt: P53_HUMAN; PIK3CA: RefSeq:NM_006218, Ensembl: ENST00000263967; CCDS:CCDS43171; UniProt: PK3CA_HUMAN. [file 13058_2020_1345_MOESM3_ESM.tif]

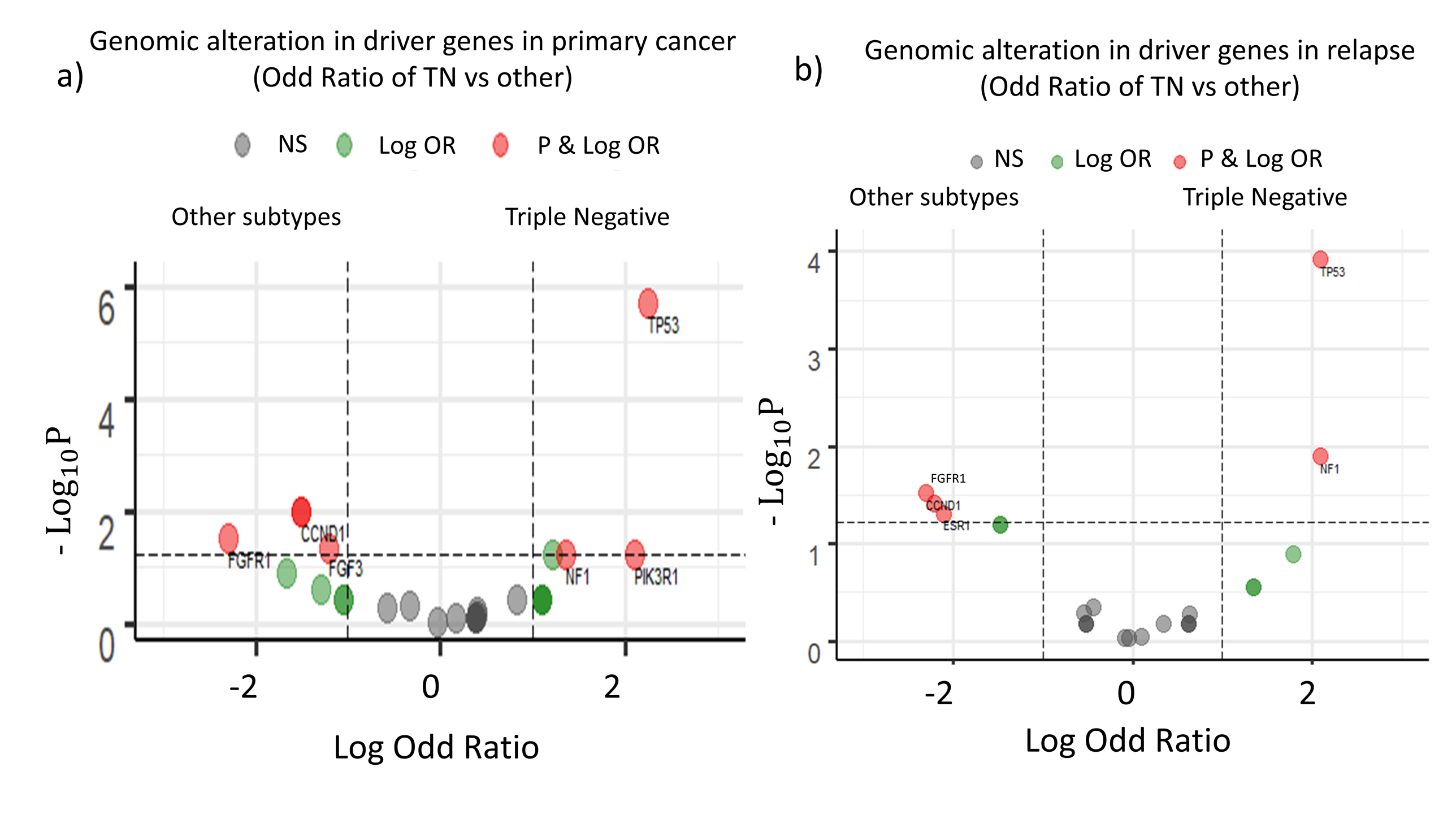

Supplement: Supplementary file 4 — Additional file 4: Supplementary Figure 3.jpg: Volcano plots of the distribution of gene driver alterations according to molecular subtype. A) Primary tumors (n = 106), B) Relapses (n = 82). LUM: non-TN tumor; TN: triple negative tumor. Green dot: genes differentially affected by alteration according to OR (OR < 0.36 or > 2.71;); Red dot: genes differentially affected by alteration according to OR and p value (OR < 0.36 or > 2.71; p value < 0.05). OR: Odds Ratio. [file 13058_2020_1345_MOESM4_ESM.tif]

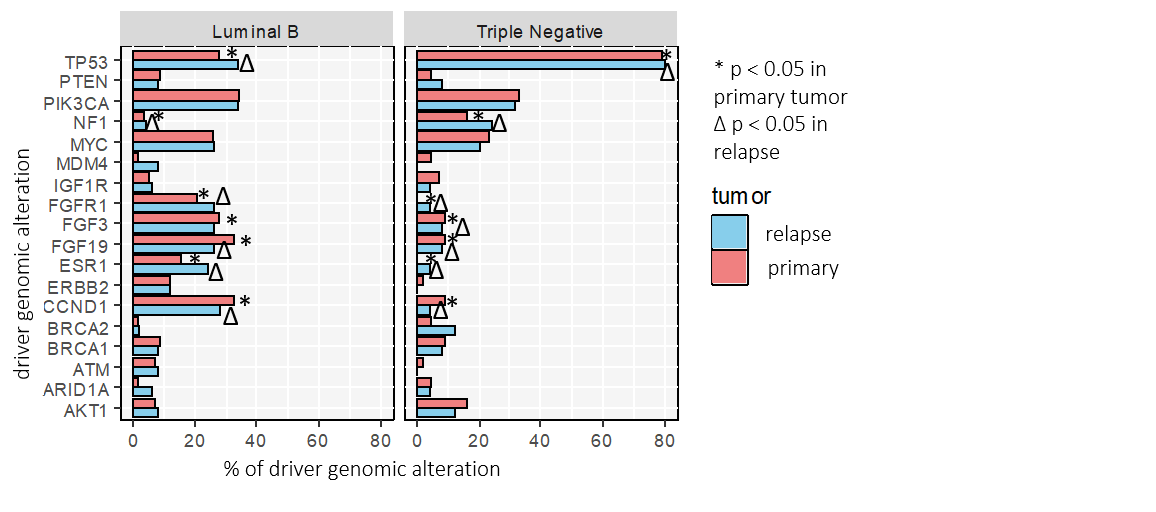

Supplement: Supplementary file 5 — Additional file 5: Supplementary Figure 4.jpg: Molecular alterations found in primary tumor and recurrent samples, according to molecular subtype. A) Primary tumors (n = 106), B) Relapses (n = 82). [file 13058_2020_1345_MOESM5_ESM.tif]

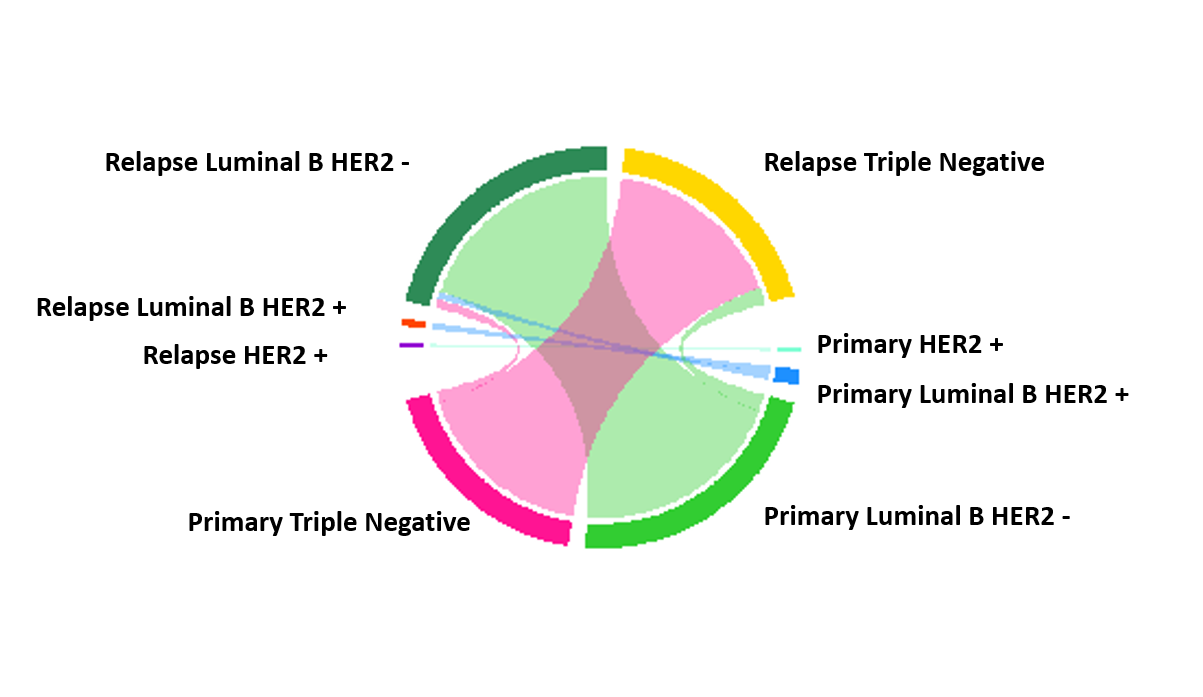

Supplement: Supplementary file 6 — Additional file 6: Supplementary Figure 5.jpg: Chord diagram representing molecular subtype change (IHC surrogates) between primary tumors and matched relapses. [file 13058_2020_1345_MOESM6_ESM.tif]

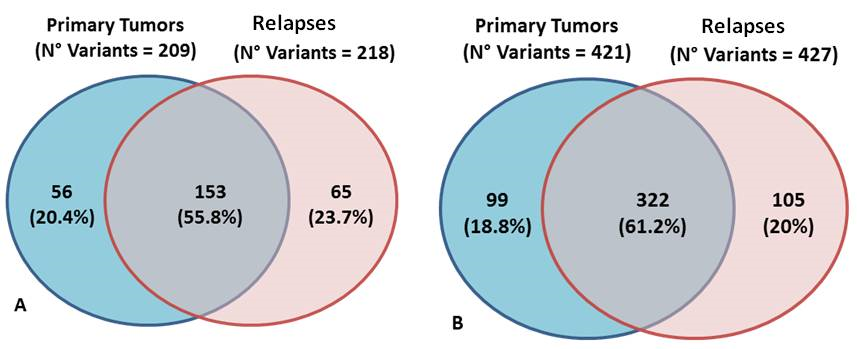

Supplement: Supplementary file 7 — Additional file 7: Supplementary Figure 6.jpg: Venn diagrams of shared and private genomic alterations between paired primary tumor and relapse samples. For each case (n = 61) primary tumor and matched recurrence were compared, considering unique and shared alterations. A) Driver alterations only, B) Driver and VUS alterations. [file 13058_2020_1345_MOESM7_ESM.tif]

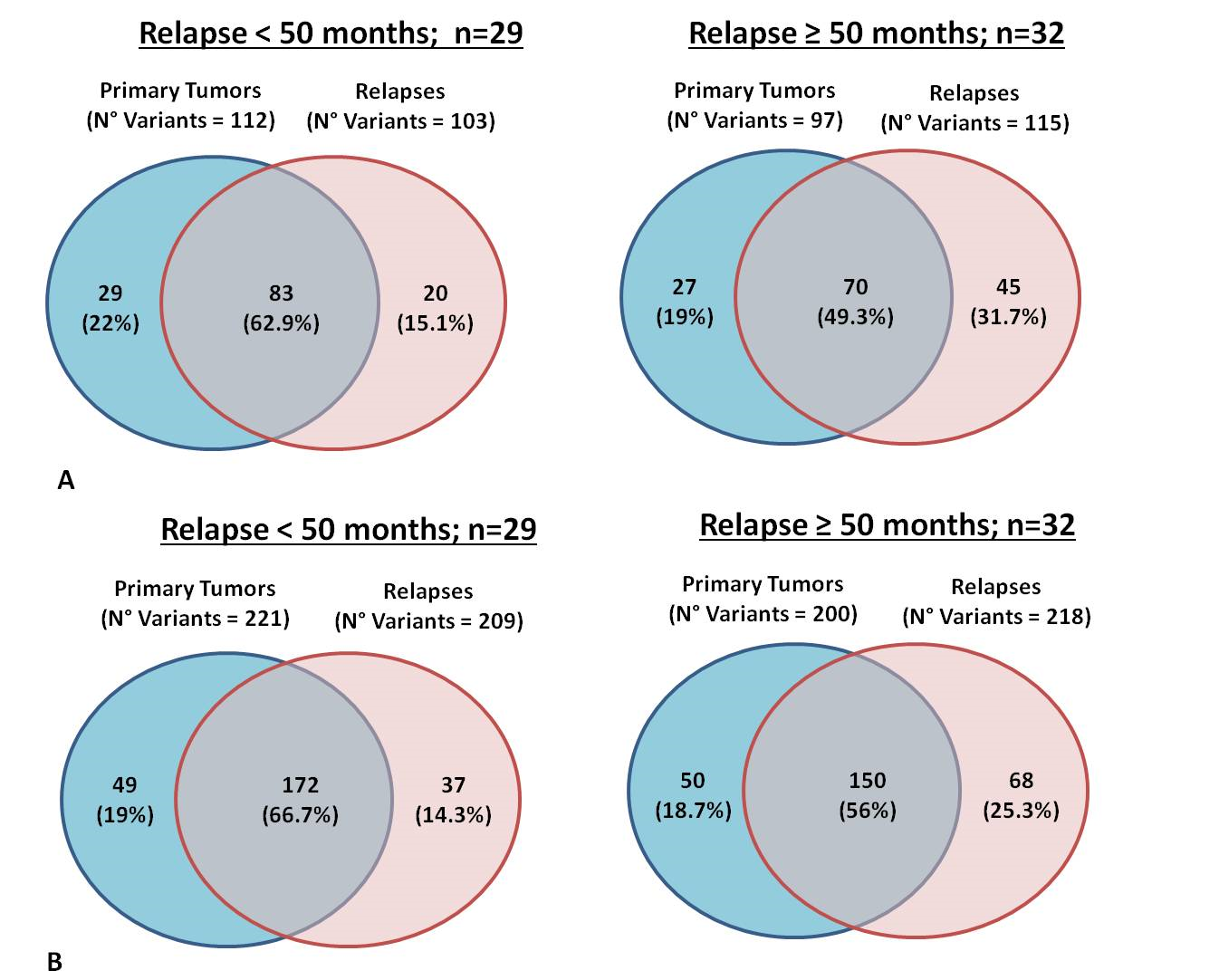

Supplement: Supplementary file 8 — Additional file 8: Supplementary Figure 7.jpg: Venn diagrams of shared and private genomic alterations between paired primary tumor and relapse samples according to time to relapse. For each case (n = 61) primary tumor and matched recurrence were compared, considering unique and shared alterations. The median time to relapse in the series (50 months) was considered as cut-off. A) Driver alterations only, B) Driver and VUS alterations. [file 13058_2020_1345_MOESM8_ESM.tif]

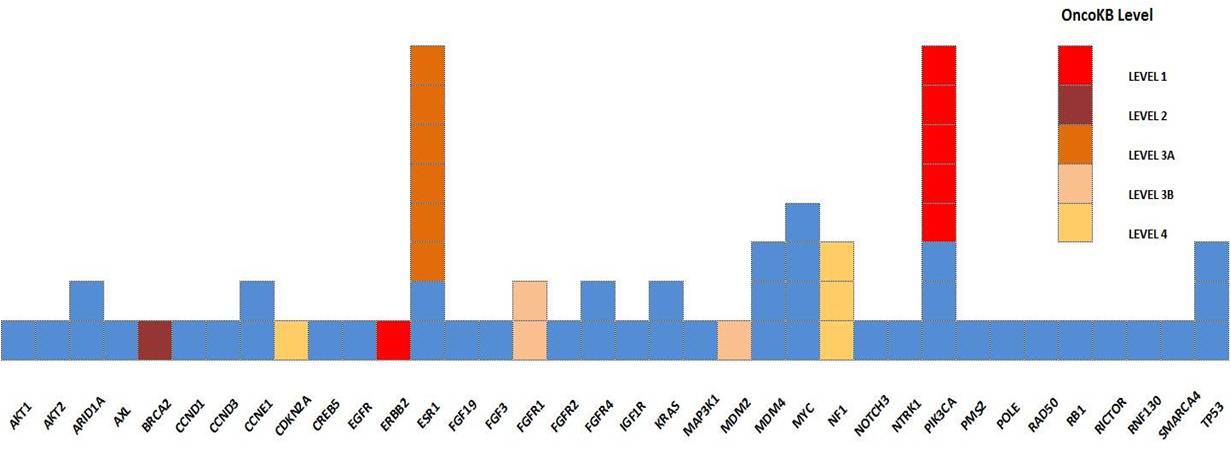

Supplement: Supplementary file 9 — Additional file 9: Supplementary Figure 8.jpg: Distribution of gene with additional driver alterations identified in recurrence samples only. Genes with additional alterations in the relapse samples and the number of additional private alteration per gene were reported on X-axis and Y-axis, respectively. Each private alteration was color-coded according to OncoKB levels as reported in the legend. Blue box: no OncoKB level associated with the specific driver alteration. [file 13058_2020_1345_MOESM9_ESM.tif]

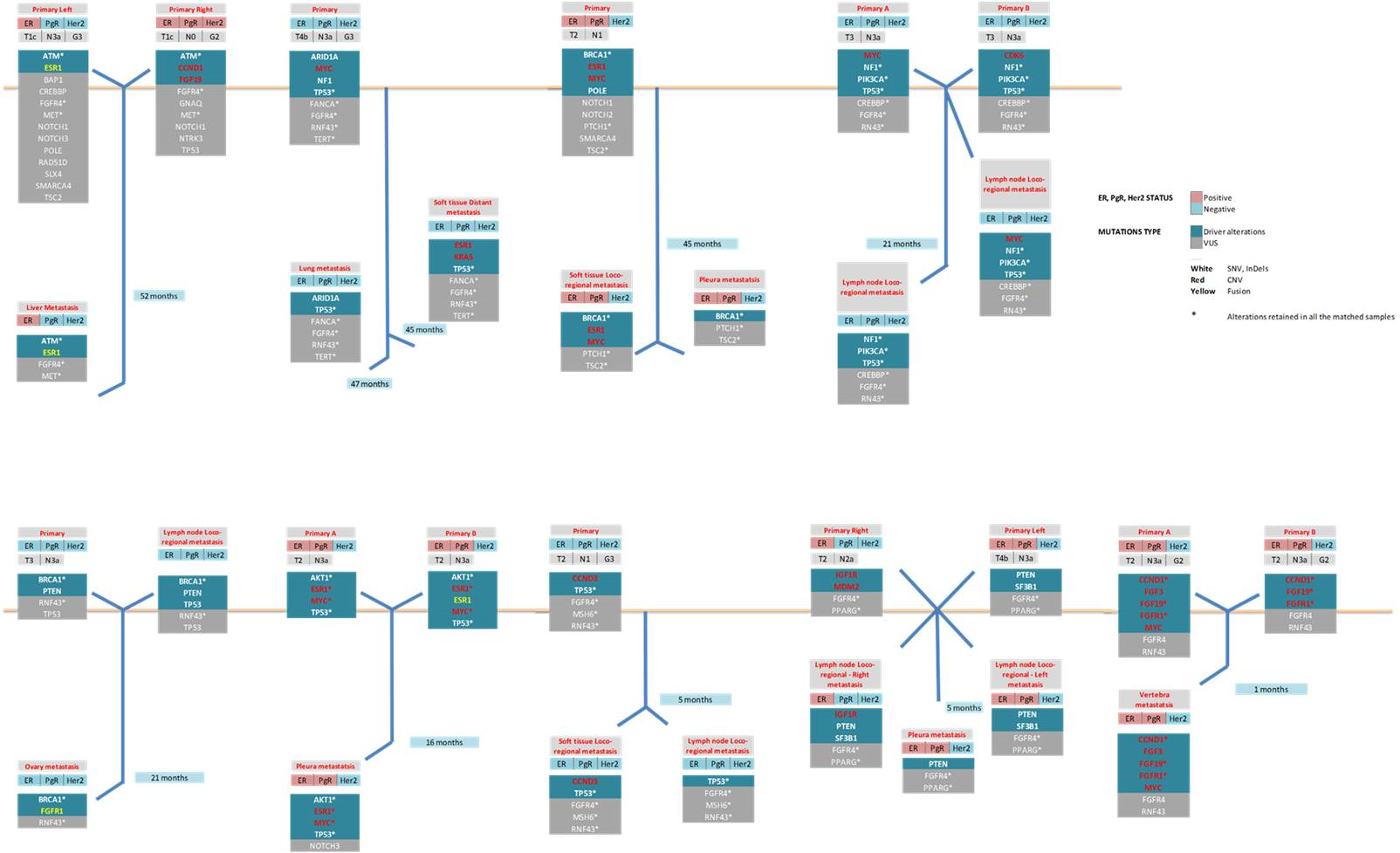

Supplement: Supplementary file 11 — Additional file 11: Supplementary Figure 9.jpg:: Graphical representation of 9 cases with multiple primary tumors and/or matched relapses. Each tree represented an individual patient. The hormone receptors status was reported for each primary tumor and relapse and color-coded according to the legend. Moreover, TNM staging and site were also indicated for each primary tumor and relapse, respectively. The time to relapse was depicted as a bar. Molecular findings were reported for each sample and color-coded as represented in the legend. Gene alterations shared between all the samples of the same patients were labeled with * mark. [file 13058_2020_1345_MOESM11_ESM.tif]
