# Supplementary material for: Inter-tumor genomic heterogeneity of breast cancers: comprehensive genomic profile of primary early breast cancers and relapses
Source: Breast Cancer Res. 2020 Oct 15;22:107. doi: 10.1186/s13058-020-01345-z (PMC7566144; doi:10.1186/s13058-020-01345-z)
Supplement: Supplementary file 10 — Additional file 10: Supplementary Table 2.docx: Additional alterations found in the recurrence samples only. Bold: driver alterations. [file 13058_2020_1345_MOESM10_ESM.docx]

**Additional file 10 - Supplementary Table 2:** Additional genomic alterations detected in relapse samples only.

| **Gene** | **N°** | **Type** | **Significance** |
| --- | --- | --- | --- |
| **AKT1** | **1** | **CNV** | **DRIVER** |
| **AKT2** | **1** | **CNV** | **DRIVER** |
| AR | 2 | SNV (1), indel (1) | VUS |
| **ARID1A** | **2** | **SNV (1), indel (1)** | **DRIVER** |
| ATRX | 1 | SNV | VUS |
| **AXL** | **1** | **CNV** | **DRIVER** |
| AXL | 1 | FUSION | VUS |
| BAP1 | 1 | SNV | VUS |
| BRCA1 | 1 | SNV | VUS |
| **BRCA2** | **1** | **indel** | **DRIVER** |
| BRCA2 | 1 | SNV | VUS |
| **CCND1** | **1** | **CNV** | **DRIVER** |
| **CCND3** | **1** | **CNV** | **DRIVER** |
| **CCNE1** | **2** | **CNV** | **DRIVER** |
| CDKN1B | 1 | SNV | VUS |
| **CDKN2A** | **1** | **SNV** | **DRIVER** |
| CDKN2B | 1 | SNV | VUS |
| **CREB5** | **1** | **FUSION** | **DRIVER** |
| CREPPB | 1 | SNV | VUS |
| **EGFR** | **1** | **CNV** | **DRIVER** |
| **ERBB2** | **1** | **CNV** | **DRIVER** |
| ERBB2 | 2 | FUSION (1), SNV (1) | VUS |
| **ESR1** | **8** | **CNV (2), SNV (5), FUSION (1)** | **DRIVER** |
| FANCD2 | 1 | SNV | VUS |
| **FGF19** | **1** | **CNV** | **DRIVER** |
| **FGF3** | **1** | **CNV** | **DRIVER** |
| **FGFR1** | **3** | **FUSION (1), CNV (2)** | **DRIVER** |
| FGFR1 | 1 | SNV | VUS |
| **FGFR2** | **1** | **CNV** | **DRIVER** |
| FGFR3 | 1 | SNV | VUS |
| **FGFR4** | **2** | **CNV** | **DRIVER** |
| FGFR4 | 1 | SNV (1) | VUS |
| H3F3A | 1 | SNV | VUS |
| HRAS | 1 | SNV | VUS |
| **IGF1R** | **1** | **CNV** | **DRIVER** |
| JAK3 | 1 | SNV | VUS |
| **KRAS** | **2** | **CNV** | **DRIVER** |
| **MAP3K1** | **1** | **SNV** | **DRIVER** |
| **MDM2** | **1** | **CNV** | **DRIVER** |
| **MDM4** | **3** | **CNV** | **DRIVER** |
| **MYC** | **4** | **CNV** | **DRIVER** |
| MYCL | 1 | SNV | VUS |
| **NF1** | **3** | **SNV(1), indel (2)** | **DRIVER** |
| NF1 | 2 | SNV | VUS |
| **NOTCH3** | **1** | **indel** | **DRIVER** |
| NOTCH3 | 1 | SNV | VUS |
| **NTRK1** | **1** | **CNV** | **DRIVER** |
| NTRK3 | 3 | CNV (1), SNV (2) | VUS |
| PDGFRA | 1 | SNV | VUS |
| **PIK3CA** | **8** | **FUSION (2), SNV (6)** | **DRIVER** |
| **PMS2** | **1** | **SNV** | **DRIVER** |
| **POLE** | **1** | **SNV** | **DRIVER** |
| POLE | 2 | SNV | VUS |
| PTCH1 | 2 | SNV | VUS |
| PTEN | 1 | SNV | VUS |
| **RAD50** | **1** | **SNV** | **DRIVER** |
| **RB1** | **1** | **indel** | **DRIVER** |
| **RICTOR** | **1** | **CNV** | **DRIVER** |
| RICTOR | 1 | SNV | VUS |
| **RNF130** | **1** | **FUSION** | **DRIVER** |
| RNF43 | 1 | SNV | VUS |
| SETD2 | 1 | SNV | VUS |
| **SMARCA4** | **1** | **SNV** | **DRIVER** |
| SMARCA4 | 1 | SNV | VUS |
| SRC | 1 | SNV | VUS |
| STK11 | 1 | SNV | VUS |
| **TP53** | **3** | **SNV (2), indel (1)** | **DRIVER** |
| XPO1 | 2 | SNV | VUS |

Bold: driver alterations
